# Essentials of Diagnosis

**Published:** 1892-10

**Authors:** 


ESSENTIALS OF DIAGNOSIS; arranged in the form of questions and answers prepared especially for students of medicine. By Solomon Solis-Cohen, M. D., of Philadelphia Polyclinic, and Aug. A. Eshner, M. D. With fifty-five illustrations, colored and otherwise. Pages, 382. Price, net, $1.50. 1892. Philadelphia: W. B. Saunders, 913 Walnut st.

This book, as its title states, is intended especially for medical students. It is elementary in character, accurate and brief. A great deal is put in a small space. B.

